# The Selective HDAC6 Inhibitor ACY-738 Impacts Memory and Disease Regulation in an Animal Model of Multiple Sclerosis

**DOI:** 10.3389/fneur.2019.00519

**Published:** 2019-06-28

**Authors:** Patrizia LoPresti

**Affiliations:** Department of Psychology, University of Illinois at Chicago, Chicago, IL, United States

**Keywords:** multiple sclerosis, drug therapy, memory, neurodegeneration, disability, CNS repair

## Abstract

Multiple sclerosis (MS) is a complex disease characterized by autoimmune demyelination and progressive neurodegeneration. Pathogenetic mechanisms of the disease remain largely unknown. Changes in synaptic functions have been reported; however, the significance of such alterations in the disease course remains unclear. Furthermore, the therapeutic potential of targeting synapses is not well-established. Synapses have key signaling elements that regulate intracellular transport and overall neuronal health. Histone deacetylase (HDAC)6 is a microtubule-associated deacetylase. The interaction between HDAC6 and microtubules is augmented by HDAC6 inhibitors. In this study, experimental autoimmune encephalomyelitis (EAE) mice, an animal model of MS, were treated with the HDAC6 inhibitor drug ACY-738 (20 mg/kg) on day 9 and day 10 post-immunization. Mice were assessed for working memory using the cross-maze test at 10 days post-immunization (d.p.i.), whereas disease scores were recorded over approximately 4 weeks post-immunization. We observed that ACY-738 delayed disease onset and reduced disease severity. Most importantly, ACY-738 increased short-term memory in a manner sensitive to disease severity. We induced EAE disease with various amounts of myelin oligodendrocyte glycoprotein (MOG35-55). EAE mice receiving 100 μg of MOG35-55 and treated with ACY-738 had a statistically significant increase in short term-memory compared to naive mice. Additionally, EAE mice receiving 50 μg MOG35-55 and treated with ACY-738 had a statistically significant increase in short term-memory when compared to EAE mice without drug treatment. In contrast, ACY-738 did not change short-term memory in EAE mice immunized with 200 μg of MOG35-55. Because ACY-738 increases short-term memory only with lower amounts of EAE-inducing reagents, we hypothesize that the inflammatory-demyelinating environment induced by higher amount of EAE-inducing reagents overpowers (at day 10 post-immunization) the synaptic molecules targeted by ACY-738. These studies pave the way for developing ACY-738-like compounds for MS patients and for using ACY-738 as a probe to elucidate disease-sensitive changes at the synapses occurring early in the disease course.

## Introduction

Multiple sclerosis (MS) is a central nervous system (CNS) neurodegenerative disease. The causes of this devastating disease are largely unknown, although autoimmune demyelination and brain inflammation are considered pivotal in the CNS damage that occurs throughout the disease course. In both MS and experimental autoimmune encephalomyelitis (EAE) (an animal model of MS), there are changes in synaptic transmission and function (1, 2) linked to the neurodegeneration, which eventually emerges during the disease with devastating clinical outcomes. Ziehn et al. (3) described deficits in memory function at 40 days post-immunization (d.p.i.) in EAE mice during the chronic form of the disease. Acharjee et al. (4) described emotional and cognitive deficits in chronic EAE during the presymptomatic stage, between 6 and 8 d.p.i. Further, LoPresti (5) identified subclinical, progressive memory decline in the relapsing-remitting (RR) EAE. Indeed, in this model, memory function was not significantly different among groups; however, memory decline occurred over time, with an initial apparent improvement in memory function as early as 10 d.p.i. Although memory function progressively declined, mobility impairment recovered, suggesting that the disease has both progressive and remitting components. Overall, such studies have elucidated that changes in synaptic transmission occur at a relatively early stage during the disease, often subclinically; such early changes may eventually be responsible for late neurodegeneration (6).

The cytoskeleton at the synapse has received attention for its role in synaptic plasticity regulation and various neuropsychiatric diseases (7). At the synapse, key functional interactions involve tubulin, end-binding proteins (EBs), Ankyrin, and actin (8). Such protein-protein interactions at the synapse regulate synaptic function and plasticity. Histone deacetylase (HDAC)6 is a microtubule-associated deacetylase (9), and such protein-protein interaction increases with administration of HDAC6 inhibitors. HDAC6 inhibitors also promote the interaction of HDAC6 with EBs (10).

HDACs are a class of enzymes targeting both histone and non-histone substrates. Non-histone substrates include transcription factors, cytoskeletal proteins, metabolic enzymes, and chaperones (11). HDAC classes consist of 18 types. HDAC6 is localized predominantly in the cytoplasm and does not deacetylate histones *in vivo* (11). The main substrate for HDAC6 is α-tubulin, although additional substrates have been identified. Such substrates include Hsp90 (heat shock protein 90) (12), cortactin (cortical actin binding protein) (13), and beta-catenin (14). Beta-catenin regulates cell–cell adhesion and gene transcription.

*In vivo* treatment with HDAC6 inhibitors increases brain α-tubulin acetylation, with no changes in acetylation levels of histones (15). Although the loss of HDAC6 does not cause toxicity, apoptosis, or major neurodevelopmental defects in rodents, it causes an antidepressant-like phenotype and memory deficits (16–19).

In this study, we analyzed EAE mice after treatment for only 2 days with the HDAC6 inhibitor ACY-738 and observed that ACY-738 delayed disease onset and attenuated disease severity. In addition, we observed that short-term memory in the cross-maze test was improved in EAE mice treated with the drug at 9 and 10 d.p.i. and tested at 10 d.p.i. Such effect was sensitive to the amount of reagent used to induce the disease.

## Materials and Methods

### EAE Induction

To induce EAE, we used an emulsion obtained from Hooke Lab (EK-0111, Hooke Kit™) and Pertussis toxin (#10033-540, Enzo Life Sciences; VWR). The emulsion from Hooke lab (see Supplementary Table 1A) contained ~1 mg/mL of myelin oligodendrocyte glycoprotein (MOG35-55) and ~5 mg/mL of killed *Mycobacterium tuberculosis* H37/Ra (MT). We injected the emulsion at volumes of 200, 100, and 50 μL. Thus, 200 μL contained 200 μg of MOG35-55 and 1 mg of MT, 100 μL contained 100 μg of MOG35-55 and 0.5 mg of MT, and 50 μL contained 50 μg MOG35-55 and 0.250 mg MT. Pertussis toxin (200 ng/100 μL/mouse) remained constant for all experiments and was injected intraperitoneally (ip) on the day of immunization and 2 days later. With higher amounts of reagents, we observed a more severe form of the disease, with a persistent severe disease score above two at 3 weeks post-immunization. With lower amounts of reagents, most of the mice recovered from a severe disease score above two. The mice were examined for ~4 weeks post-immunization. The amounts used in this study to induce chronic (CH) vs. relapsing-remitting (RR)-EAE are included in Supplementary Table 1A, together with a summary of previous work showing various concentrations of the reagents used to induce either CH- or RR-EAE (Supplementary Table 1B).

C57BL/6 female mice between 7 and 8 weeks of age were ordered from Jackson Laboratory and housed for 1 week before EAE induction. Mice were immunized subcutaneously (sc) (200 μL/mouse) with 200 μg/mouse of MOG35–55 peptide emulsion in complete Freund's adjuvant (CFA) (EK-0111, Hooke Kit™). Experiments were also performed with volumes of 100 μL/mouse and 50 μL/mouse (from kit EK-0111, Hooke Kit™). Pertussis toxin (200 ng/100 μL/mouse) remained constant for all experiments and was injected ip on the day of immunization and 2 days later. EAE mice were graded on a scale of 0–5: 0, no disease; 1, limp tail; 2, hind limb weakness; 3, one or two hind limb paralysis; 4, hind and fore limb paralysis; and 5, moribund and death (5). Disease scores were the averages obtained at each time point from five mice/group/experiment. Mean disease scores (±SEM) were calculated from these disease scores. We collected 44 disease scores per group from seven experiments.

### Drug Treatment

ACY-738 powder (Celgene Corporation, Acetylon Pharmaceuticals) was dissolved in dimethyl sulfoxide (DMSO) and diluted in phosphate-buffered saline (PBS) for ip injection of 200 **μ**L (20 mg/kg) on days 9 and 10 post-immunization. The drug was injected on day 9 (~1:00 p.m.) and day 10 (~12:00 p.m.) post-immunization; mice were tested in the cross-maze test on day 10 post-immunization. The EAE mice treated with the drug (EAE+ D) were tested starting 1 hour and 30 min after the last drug injection.

### Cross-Maze Exploration Test

The Cross-maze exploration test was performed to evaluate spatial working memory using a protocol described previously (5). Briefly, each mouse was placed in the center of a four-arm cross-maze apparatus and was permitted to enter each arm freely (each arm was marked A, B, C, or D). Each mouse was evaluated for up to 31 entries. An entry occurred when all four paws entered the arm. An alternation occurred when an entry occurred into each of the four distinct arms (e.g., A, D, C, B, or C, D, A, B; but not D, A, C, A). Percentage of alternation was used as an indicator of memory strength, when successive entries took place into the four arms in overlapping quadruple sets. Data are indicated as percent alternation, an indicator of short-term memory. Percent alternation value is equal to the ratio of actual/possible alternations × 100 (5). Data are presented as mean ± SEM in the **Table 2**, and are presented as mean ± SE in the corresponding histogram.

### Statistical Analysis

Each experiment comprised five mice/group. Disease scores were the averages calculated from five mice per group at distinct times. Forty-four disease scores were collected per group and from seven independent experiments. Mean disease scores (±SEM) were calculated from the disease scores. Mean disease scores (±SEM) were compared with independent samples *t*-test. We measured mean disease scores between 11 and 14, 15 and 18, and 19 and 32 d.p.i. In Table 1, **“***n*” represents the number of disease scores obtained over time and from distinct experiments. In addition to independent samples *t*-test, statistical analysis was performed using mixed effects linear regression model. Clustering of observations within experiments (ICC = 0.46, *z* = 2.01, *p* = 0.0224) was accounted for with a random intercept term.

Table 1**(A)** Disease score analysis with *t-*test and **(B)** Disease score analysis with mixed effects linear regression model.

**Table:** 

**(A)**					
**Treatment**	**Disease score ≥ 1.5**	**Mean disease score ± SEM at 11–14 d.p.i**.	**Mean disease score ± SEM at 15–18 d.p.i**.	**Mean disease score ± SEM at 19–32 d.p.i**.	**Cumulative disease score**
EAE	25/44	1.160 ± 0.248	1.989 ± 0.205	1.657 ± 0.220	76.4
EAE + D	7/44	0.36 ± 0.160	0.989 ± 0.114	0.857 ± 0.175	37.6
		*n* = 5; *p* = 0.0267^**^	*n* = 18; *p* = 0.0001^**^	*n* = 21; *p* = 0.0069^**^	*n* = 44

**Table:** 

**(B)**					
	**Disease score**				***t*****-test for EAE vs. EAE** **+** **D**
	**Mean**	**SE**	**95% C.I**.		*t* = 2.40, do = 74, *p* = 0.0188
EAE	1.670	0.197	1.241	2.100	
EAE + D	1.000	0.197	0.570	1.430	

**Figure 1:**
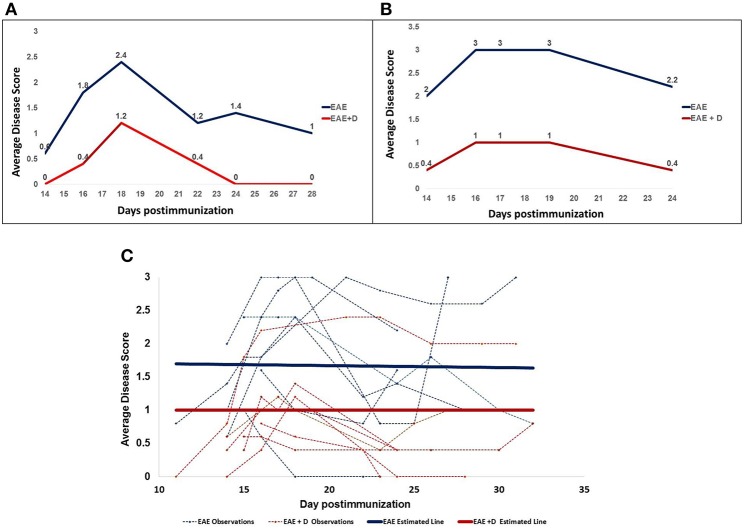
The selective HDAC6 inhibitor ACY-738 regulates experimental autoimmune encephalomyelitis in both Relapsing-Remitting **(A)** and Chronic **(B)** EAE mice. **(C)** Shows all disease scores and an estimated line for the disease scores.

*The drug administered on days 9 and 10 post-immunization (20 mg/kg) reduced disease severity in both Relapsing-Remitting (RR) **(A)** and Chronic (CH) **(B)** EAE mice. Figures 1A,B have the disease scores. Each disease score is the average obtained from five mice/group, in blue for EAE mice and in red for EAE + D mice. Figure 1C shows all disease scores collected from seven experiments at distinct times, together with an estimated line for the disease scores of EAE (blue) and EAE + D (red) mice*.

*In **(A)**, n represents the number of disease scores. Among the 44 disease scores per group, EAE mice had twenty-five over 1.5, whereas EAE + D mice had only seven over 1.5. **(A)** has mean disease scores (± SEM) during early (11–14 d.p.i.), mid phase (15–18 d.p.i.), and at the end of disease course (19–32 d.p.i.) with statistically significant differences in EAE vs. EAE + D mice over the course of the entire disease. The cumulative disease score (from all the disease scores) shows also an overall decrease in disease severity of about 50% in EAE+D mice. **(B)** displays the statistical analysis with a linear regression model and statistically significant differences between EAE and EAE + D mice (Mean = 1,670 and 1,000, respectively; p = 0.0188). n indicates the number of disease scores. Disease score is the average obtained from five mice/group*.

** *p < 0.05*.

For the cross-maze test, we applied independent samples *t*-test and one-way ANOVA. At each dosage level, one-way ANOVA with **two** degrees of freedom was used to test the null hypothesis of equal means across all the three groups (naïve, EAE, and EAE + D). Pairwise comparisons were made using independent samples *t*-test and the more conservative Tukey's test. One-way ANOVA was used to compare the overall mean response across the three dosage levels. For the independent samples *t*-test, we used GraphPad QuickCalcs online program. For one-way ANOVA and mixed effects linear regression model, we used the PROC ANOVA in SAS version 9.4.

For disease scores, *p* < 0.05 was considered statistically significant using the independent samples *t*-test. For behavioral test, *p* < 0.05 was considered statistically significant using the independent samples t-test and one-way ANOVA (^**^p < 0.05). One asterisk (^*^p < 0.05, independent samples t-test) denotes *p* < 0.1 with one-way ANOVA.

## Results

### The Selective HDAC6 Inhibitor ACY-738 Regulates Experimental Autoimmune Encephalomyelitis Disease

Drug administration on days 9 and 10 post-immunization (20 mg/kg) reduced disease severity in both RR and CH EAE. Representative examples are provided in Figure 1A for RR-EAE and in Figure 1B for CH-EAE. Disease score was the average calculated from five mice/group, indicated in blue for EAE mice and in red for EAE + D mice (Figures 1A,B).

Disease scores collected at distinct times over ~4 weeks post-immunization were obtained from seven independent experiments. Disease score was the average calculated from five mice/group at a specific time and from distinct experiments. The experiments included both RR- and CH-EAE disease. Table 1A shows that of the 44 disease scores, twenty-five disease scores were higher than 1.5 in EAE mice; whereas only seven disease scores were higher than 1.5 in EAE + D mice. In addition, we calculated mean disease scores (±SEM) from disease scores taken at various times during the disease and from independent experiments. Early in the disease (11–14 d.p.i.), mean disease score was 1.160 ± 0.248 in EAE mice vs. 0.360 ± 0.160 in EAE + D mice, with a statistically significant difference of *p* = 0.0267 (*n* = 5, where *n* indicates the number of disease scores). During the mid phase of the disease (15–18 d.p.i.), mean disease score was 1.989 ± 0.205 in EAE mice vs. 0.989 ± 0.114 in EAE + D mice, with a statistically significant difference of *p* = 0.0001 (*n* = 18). At the end of disease course (19–32 d.p.i.), mean disease score was 1.657 ± 0.220 in EAE mice vs. 0.857 ± 0.175 in EAE + D mice, with a statistically significant difference of *p* = 0.0069 (*n* = 21). Thus, the difference between untreated and treated groups reached statistical significance (independent samples *t*-test) over the entire course of the disease. In addition, by combining all the disease scores collected from the various experiments at various times, the cumulative disease score was 76.4 in EAE mice vs. 37.6 in EAE + D mice, which showed an overall reduction in disease severity of about 50%.

In addition, mixed effects linear regression model revealed that the effects of treated vs. untreated was −0.67 (*p* = 0.0188), indicating that the disease score was 0.67 less in the treated animals than in the untreated animals at any time point. Estimated means from the linear regression model and results of the independent samples *t*-test of the main effects indicate a statistically significant reduction in disease score with treatment (*p* = 0.0188). The estimates from the model accounted for the clustering of repeated measures, whereas the independent samples *t*-test assumed each of the two compared groups were a set of independent observations. In contrast, the estimated slope in EAE mice was −0.003 (*p* = 0.8471), whereas in EAE + D mice, it was 0.00 (*p* = 1.000). The two parallel lines across time for EAE and EAE + D mice had a common slope of −0.0015 (*p* = 0.8907), indicating a slight decrease that was not statistically significant. Thus, the slope was the same in both groups, suggesting that the disease, although diminished in its severity secondary to drug treatment, was not altered in its dynamics; i.e., the disease displayed similar trends in EAE vs. EAE + D mice, although EAE + D had significantly lower disease scores (Figure 1C, Table 1B).

Notably, drug treatment delayed disease onset. Disease onset occurred between 11 and 14 d.p.i. Figure 1A shows that in RR-EAE +D mice, the disease had not yet started at 14 d.p.i., whereas EAE mice with no drug treatment already exhibited mobility defects revealed by disease scores above zero. Such delay in disease onset was striking when a high dose (50 mg/kg) of a single drug injection was administered at 10 d.p.i. (Supplementary Material). In this experiment conducted with five mice per group, differences were evident at 11 d.p.i. (24 hours post-treatment), suggesting that the drug abruptly halted the disease.

### The Selective HDAC6 Inhibitor ACY-738 Regulates Short-Term Memory in a Manner Sensitive to Disease Severity

**Figure 2:**
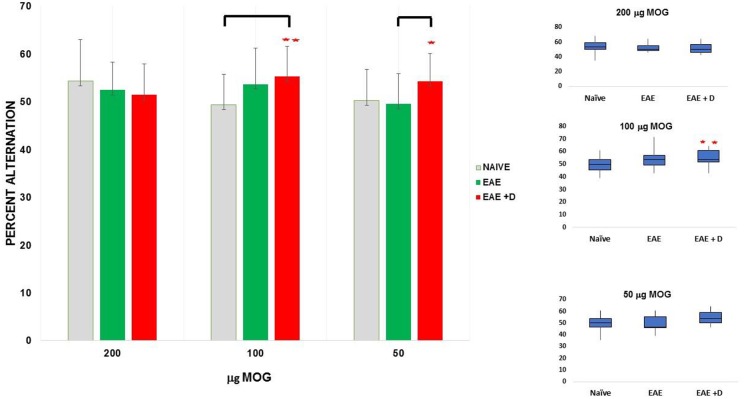
The selective HDAC6 inhibitor ACY-738 regulates short-term memory in a manner sensitive to disease severity. ^**^p < 0.05 independent samples t-test and one-way ANOVA. ^*^p < 0.05 independent samples t-test.

We measured short-term memory with the cross-maze test at day 10 post-immunization. We combined the data from three independent experiments performed with mice receiving 200 μg MOG35-55. No significant differences among the groups were observed. We combined the data from four independent experiments performed with 100 μg MOG35-55. A statistically significant difference between Naïve and EAE + D groups was observed. We combined the data from three independent experiments performed with 50 μg MOG35-55. A statistically significant difference between EAE and EAE + D groups was noted (Figure 2 and Table 2).

**Table 2:** The selective HDAC6 inhibitor ACY-738 regulates short-term memory in a manner sensitive to disease severity.

**MOG35-55**	**NAÏVE (N)**		**EAE (E)**		**EAE** **+** **D (ED)**		***p*****-values**			
		***t* test**		***t* test**		***t* test**				
**μg**	***n***	**mean ± SEM**	***n***	**mean ± SEM**	***n***	**mean ± SEM**	**N vs. E**	**N vs. ED**	**E vs. ED**	**One-way ANOVA**
200	15	54.3 ± 2.2	15	52.4 ± 1.5	15	51.4 ± 1.8	0.4854	0.3335	0.6950	0.5569
100	20	49.3 ± 1.4	20	53.6 ± 1.7	19	55.3 ± 1.5	0.0613	0.0058^**^	0.4562	0.0234^**^
50	15	50.2 ± 1.7	15	49.5 ± 1.6	15	54.3 ± 1.5	0.7633	0.0828	0.0396^*^	0.0890

*Naïve mice were not administered any drug. EAE was induced with various amounts of EAE-inducing reagents (200, 100, and 50 μg MOG35-55). Data are presented as mean ± SEM in the Table, and as mean ± SE in the corresponding histogram. Comparison with independent samples t-test revealed that in EAE mice administered 100 μg MOG35-55, the difference between Naïve and EAE + D mice was statistically significant (p = 0.0058). This difference was also statistically significant at α = 0.05 using Tukey's studentized range test. Comparison with independent samples t-test revealed that in mice receiving 50 μg MOG35-55, the difference between EAE and EAE + D mice was also statistically significant (p = 0.0396). One-way ANOVA revealed a statistically significant difference in the group administered 100 μg MOG35-55 (F = 4.02, p = 0.0234) and in the group administered 50 μg MOG35-55 (F = 2.56, p = 0.0890). One-way ANOVA data are displayed as boxplots. In boxplots, the central black line represents the median, the bottom and top boundaries represent quartiles. n indicates the number of mice*.

** *p < 0.05 independent samples t-test and one-way ANOVA*.

* *p < 0.05 independent samples t-test*.

#### Experiments With 200 μg MOG35-55

Fifteen mice (*n* = 5 each for Naïve, EAE, and EAE + D) were used for each experiment. Each experiment was repeated three times, and the data obtained with the cross-maze test on day 10 post-immunization were combined. We observed that the difference between Naïve and EAE mice was not statistically significant (mean ± SEM, 54.3 ± 2.2 vs. 52.4 ± 1.5, respectively; *p* = 0.4854). The difference between Naïve and EAE + D mice was not statistically significant (mean ± SEM, 54.3 ± 2.2 vs. 51.4 ± 1.8, respectively; *p* = 0.3335). In addition, the difference between EAE and EAE + D mice was not statistically significant (mean ± SEM, 52.4 ± 1.5 vs. 51.4 ± 1.8, respectively; *p* = 0.6950). There were also no statistically significant differences across the means of the three groups as determined by one-way ANOVA (F = 0.59, *p* = 0.5569).

#### Experiments With 100 μg MOG35-55

Fifteen mice (*n* = 5 each for Naïve, EAE, and EAE + D) were used for each experiment. Each experiment was repeated four times, and the data were combined. In one of the experiments, only fourteen mice were analyzed (*n* = 5 each for Naïve and EAE, and *n* = 4 for EAE + D). We observed that the difference between Naïve and EAE mice was not statistically significant (mean ± SEM, 49.3 ± 1.4 vs. 53.6 ± 1.7, respectively; *p* = 0.0613); whereas the difference between Naïve and EAE + D mice was statistically significant (mean ± SEM, 49.3 ± 1.4 vs. 55.3 ± 1.5, respectively; *p* = 0.0058). Such difference was significant at α = 0.05 using Tukey's studentized range test. In contrast, the difference between EAE and EAE + D mice was not statistically significant (mean ± SEM, 53.6 ± 1.7 vs. 55.3 ± 1.5, respectively; *p* = 0.4562). One-way ANOVA, revealed a statistically significant difference between the three group means (F = 4.02, df = 2, *p* = 0.0234).

#### Experiments With 50 μg MOG35-55

Fifteen mice (*n* = 5 each for Naïve, EAE, and EAE + D) were used for each experiment. Each experiment was repeated three times, and the data were combined. We found that the difference between Naïve and EAE mice was not statistically significant (mean ± SEM, 50.2 ± 1.7 vs. 49.5 ± 1.6, respectively; *p* = 0.7633). The difference between Naïve and EAE + D mice was not statistically significant (mean ± SEM, 50.2 ± 1.7 vs. 54.3 ± 1.5, respectively; *p* = 0.0828). In contrast, the difference between EAE and EAE + D mice was statistically significant (mean ± SEM, 49.5 ± 1.6 vs. 54.3 ± 1.5, respectively; *p* = 0.0396). There was a statistically significant difference between group means as determined by one-way ANOVA (F = 2.56, df = 2, *p* = 0.0890). The contrast between EAE vs. EAE+ D was significant using a independent samples *t*-test but not under the more conservative Tukey's test. Comparison of all the data in the group with 200, 100, and 50 μg MOG35-55 revealed no statistically significant differences across the group means as determined by one-way ANOVA (F = 0.57, df = 2, *p* = 0.5665) (data not shown).

## Discussion

The positive effects of ACY-738 on disease course occurred after one or two injections, and protection occurred within 24 hours post-treatment. Work by Ren et al. (20) showed that ACY-738 decreased innate and adaptive immune responses in a model of systemic lupus erythematosus; ACY-738 reduced disease pathogenesis by altering differentiation of T and B cells (21). However, these positive effects were observed after long-term treatment lasting several weeks. We did not assess the mechanisms by which ACY-738 protects from EAE disease; however, the beneficial outcomes within 24 hours post-treatment may be related to an effect of ACY-738 on the neuronal cytoskeleton and/or secondary to a lethal, acute, effect of ACY-738 against cells attacking myelin. Indeed, it was previously shown that ACY-738 induces cell death *in vitro* when used at high concentrations (22). In addition, Guo et al. (23) reported that HDAC6 inhibition reverses axonal transport defects in motor neurons derived from FUS-ALS patients. Mutations in FUS (fused in sarcoma) cause amyotrophic lateral sclerosis (ALS). It is known that early in EAE, axonal transport deficits are present, and reduced levels of KIF5A (kinesin heavy chain isoform 5A) were reported in MS patients (6, 24, 25). Thus, part of the beneficial effects observed for the disease course could be secondary to positive regulation of axonal transport exerted by ACY-738. Indeed, the inhibition of HDAC6 may regulate both anterograde and retrograde transport due to the regulation of kinesin and dynein motors (26).

Acetylation of α-tubulin occurs at lysine 40 at the inner surface. Additional sites of acetylation have been identified in both α- and β- tubulin (27). Further studies are required to determine the functional consequences of HDAC6 inhibitors on post-translational modification of these various sites of tubulin. This information could facilitate effective pharmacological targeting of cytoskeleton dynamics at the synapse, with beneficial impacts on axonal transport regulation.

Drugs such as TSA (Trichostatin A) or SAHA (suberoyl + anilide + hydroxamic acid) inhibit both HDAC6 and class I isoforms, whereas drugs such as tubacin and tubastatin A selectively inhibit HDAC6 (11, 28, 29). Interestingly, ACY-738 is a selective inhibitor of HDAC6 and has the unique property of rapid distribution in the brain, with a short plasma half-life of 12 min (11).

Pathways that regulate synaptic plasticity are critical for brain health and prevention of neuropsychiatric and degenerative diseases (7). In this study, we developed an experimental model that can establish pharmacological targets at the synaptic cytoskeleton upon which ACY-738 acts. Further, ACY-738 will allow us to investigate how short-term memory is regulated. While the role of HDAC6 in synaptic plasticity and memory is established (30), the dynamics of cytoskeletal interactions at the synapse require additional investigation. Our model may reveal dynamic regulation at synapses that requires pharmacologic rescue to treat selective memory deficits during various diseases of the CNS.

Jochems et al. (11) reported that upon acute treatment, ACY-738 improved ambulation levels and decreased anxiety. Majid et al. (31) showed that ACY-738 improved Alzheimer's disease phenotype in amyloid precursor protein/presenilin 1 mice. In particular, this study indicated that drug administration increased cognition; however, the drug was administered for 21 and 90 days. In addition, Selenica et al. (32) showed that tubastatin A, a selective HDAC6 inhibitor, improved memory and reduced total tau levels in a mouse model of tau deposition. However, the mice were treated for 2 months. Zhang et al. (33) used tubastatin A and ACY-1215 to rescue cognitive deficits in a mouse model of Alzheimer's disease and found that both tubastatin A and ACY-1215 reduced behavioral deficits, amyloid-β load, and tau hyperphosphorylation. However, the mice were treated for 20 consecutive days; ACY-1215 is a selective HDAC6 inhibitor. In contrast, in this study, we analyzed mice after treatment with ACY-738 for only two days and observed an increase of short-term memory.

The cross-maze test relies on working memory, which depends on selected CNS areas including the hippocampus, septum, basal forebrain, and prefrontal cortex. The cytoskeleton at the synapse has a role in synaptic plasticity regulation and various neuropsychiatric diseases (7). Protein-protein interactions at the synapse regulate synaptic function and plasticity. At the synapse, key functional interactions involve tubulin, EBs, ankyrins, and actin (8). HDAC6 inhibitors increase the interaction of HDAC6 with microtubules and EBs (10). HDAC6 also regulates growth factor-induced actin remodeling and endocytosis (34); thus, HDAC6 inhibitors may also alter functional regulation of actin. Anxiety- and depression-like behaviors were described in EAE mice before any motor defect became apparent (2, 4), so our experimental conditions may have brought the antidepressive properties of ACY-738 to light (11). Finally, the positive effects on memory may be partly explained by enhancement of stress resilience through HDAC6-mediated regulation of glucocorticoid receptor chaperone dynamics (11). In this respect, additional studies are necessary to elucidate the mechanisms by which ACY-738 acts on memory regulation. Nicotine, previously shown to inhibit HDAC6 and chaperone-dependent activation of glucocorticoid receptors in cultured cells, had a neuroprotective effect in an experimental model of MS (35, 36). In summary, with the aim of developing the most effective treatments for MS patients, future studies should aim to understand similarities and differences among various inhibitors directed at HDAC6, so selective drugs of such class with the highest safety and efficacy could provide breakthrough therapy for the neurodegeneration in patients affected by MS.

## Ethics Statement

All experiments were conducted with approval of the University of Illinois Institutional Animal Care and Use Committee.

## Author Contributions

The author confirms being the sole contributor of this work and has approved it for publication.

### Conflict of Interest Statement

The author declares that the research was conducted in the absence of any commercial or financial relationships that could be construed as a potential conflict of interest.
