# Supplementary material for: The Selective HDAC6 Inhibitor ACY-738 Impacts Memory and Disease Regulation in an Animal Model of Multiple Sclerosis
Source: Front Neurol. 2019 Jun 28;10:519. doi: 10.3389/fneur.2019.00519 (PMC6609573; doi:10.3389/fneur.2019.00519)
Supplement: Supplementary file 1 [file Data_Sheet_1.docx]

**Experimental autoimmune encephalomyelitis (EAE) Induction.** To induce EAE disease, we used an emulsion purchased from Hooke lab (EK-0111, Hooke Kit™) and Pertussis toxin (#10033-540, Enzo Life Sciences; VWR). The emulsion from Hooke lab (Table 1A) contained ~1 mg/mL of myelin oligodendrocyte glycoprotein (MOG35-55) and ~5 mg/mL of killed *Mycobacterium tuberculosis* H37/Ra (MT). Volumes of 200, 100, and 50 μL were administered to the mice. Thus, 200 μL contained 200 μg of MOG35-55 and 1 mg of MT, 100 μL contained 100 μg of MOG35-55 and 0.5 mg of MT, and 50 μL contained 50 μg MOG35-55 and 0.250 mg MT. Pertussis toxin (200 ng/100 μL/mouse) remained constant for all experiments and was injected intraperitoneally (ip) on the day of immunization and 2 days later. With greater amounts of EAE-inducing reagents, EAE mice exhibited a more severe form of the disease, with a persistent severe disease score above two at 3 weeks post-immunization. With smaller amounts, most of the mice recovered from a severe disease score. The mice were examined for approximately 4 weeks post-immunization.

C57BL/6 female mice between 7-8 weeks of age were obtained from Jackson Laboratory and housed for 1 week before EAE induction. Mice were immunized subcutaneously (SC) (200 μL/mouse) with 200 μg/mouse of MOG35–55 peptide emulsion in complete Freund's adjuvant (CFA) (EK-0111, Hooke Kit™). Experiments were also performed with 100 μL/mouse and 50 μL/mouse (from kit EK-0111, Hooke Kit™). Pertussis toxin (200 ng/100 μL/mouse) volume was the same for all experiments and was injected ip on the day of immunization and 2 days post-immunization. EAE mice were graded on a scale of 0–5. 0, no disease; 1, limp tail; 2, hind limb weakness; 3, one or two hind limb paralysis; 4, hind and fore limb paralysis; and 5, moribund and death (LoPresti, 2015). Disease score for timepoints was the average obtained from five mice/condition, whereas the mean disease scores (± SEM) were calculated from the disease scores. Table 1A indicates the amounts of reagents used in this study to induce chronic (CH) vs. relapsing-remitting (RR)-EAE. Table 1B lists the amount of reagents used in other studies.

**Table 1A**

| **Disease course** | **MOG35-55 (~1 mg/mL)**  *Hooke kit EK-0111* | **Killed *Mycobacterium***  ***tuberculosis* H37/Ra (5 mg/ml)**  *Hooke kit EK-0111* | **Pertussis toxin**  *Enzo Life Science* |
| --- | --- | --- | --- |
| **CH-EAE** | 200 μg (200 μL) | 1 mg (200 μL ) | 200 ng |
| **RR-EAE** | 100 μg (100 μL) | 0.5 mg (100 μL ) | 200 ng |
| **RR-EAE** | 50 μg (50 μL) | 0.25 mg (50 μL ) | 200 ng |

**Table 1B**

| **References** | **MOG35-55** | **Killed *Mycobacterium tuberculosis*** | **Pertussis toxin** |
| --- | --- | --- | --- |
| **CH-EAE**  *Berard et al., 2009* | 300 μg | 4 mg/mL | 300 ng |
| **RR-EAE**  *Berard et al., 2009* | 50 μg | 0.5–1.0 mg/mL | 200 ng |
| **RR-EAE**  *Fife et al., 2000, LoPresti, 2015* | 200 μg | 4 mg/mL | 200 ng |

**ACY-738 Delays Experimental Allergic Encephalomyelitis Disease Onset and Reduces Disease Severity**

Disease onset occurred between 11 and 14 days post-immunization (d.p.i.). The delay of disease onset was notable with a high dose (50 mg/kg) of a single drug injection at 10 d.p.i. In this experiment (n=5 mice/group), differences were evident at 11 d.p.i. (24 hours post-treatment), suggesting that the drug abruptly halted the disease (Figure 1).

**Figure 1. ACY-738 Delays Experimental Allergic Encephalomyelitis**

**Disease Onset and Reduces Disease Severity**

**
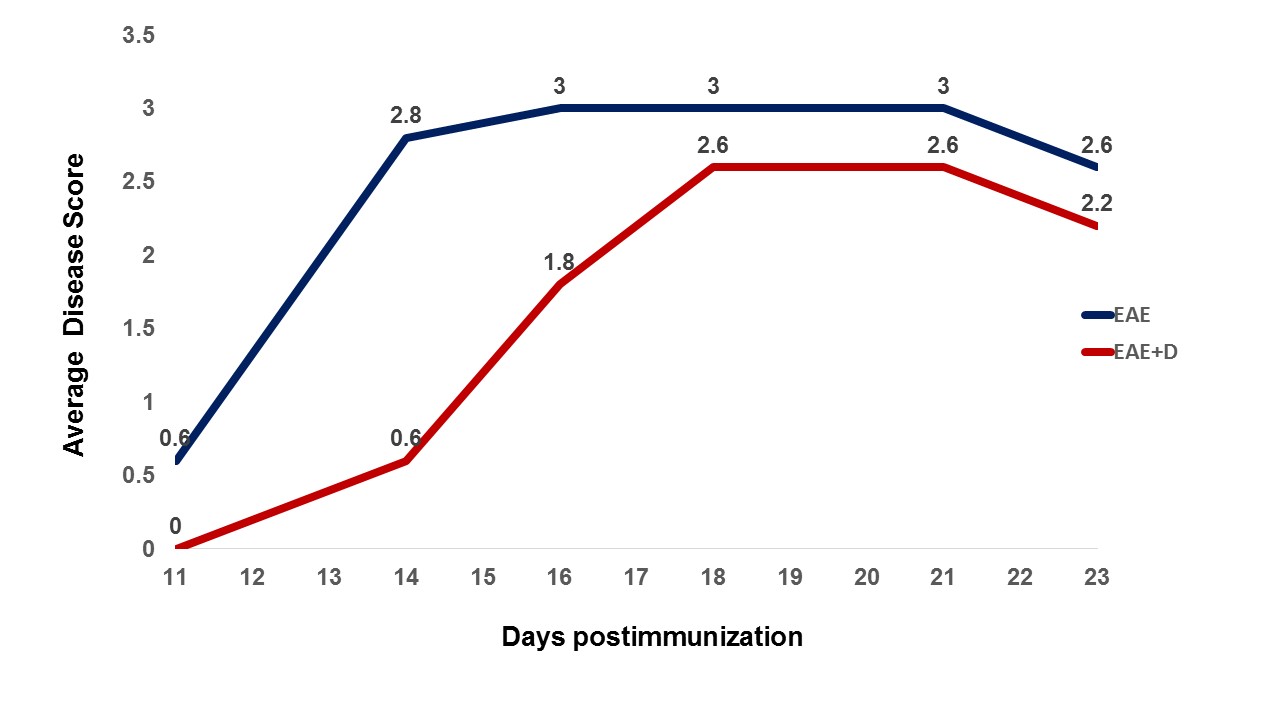
**

**Legend** Drug administration on day 10 postimmunization (50 mg/kg) delays disease onset and reduces disease severity. Figure indicates the disease scores that were calculated for five mice/group in blue for EAE mice and in red for EAE + D mice.

**References**

Berard JL, Wolak K, Fournier S, David S (2010) Characterization of relapsing-remitting and chronic forms of experimental autoimmune encephalomyelitis in C57BL/6 mice. Glia 58:434–445.

Fife BT, Huffnagle GB, Kuziel WA, Karpus WJ (2000) CC chemokine receptor 2 is critical for induction of experimental autoimmune encephalomyelitis. J Exp Med 192:899–905

LoPresti P. (2015) Glatiramer acetate guards against rapid memory decline during relapsing-remitting experimental autoimmune encephalomyelitis. Neurochem Res. 40:473–9.
